# A Perspective and Framework for Developing Sample Type Specific Databases for LC/MS-Based Clinical Metabolomics

**DOI:** 10.3390/metabo10010008

**Published:** 2019-12-21

**Authors:** Nichole A. Reisdorph, Scott Walmsley, Rick Reisdorph

**Affiliations:** 1 Department of Pharmaceutical Sciences, School of Pharmacy and Pharmaceutical Sciences, University of Colorado Anschutz Medical Campus, 12850 East Montview Boulevard, Aurora, CO 80045, USA; Richard.Reisdorph@cuanschutz.edu; 2 Masonic Cancer Center, University of Minnesota, 516 Delaware St. SE, Minneapolis, MN 55455, USA; swalmsle@umn.edu; 3 Institute for Health Informatics, University of Minnesota, 516 Delaware St. SE, Minneapolis, MN 55455, USA

**Keywords:** metabolomics, database, spectral library, compound identification, metabolite identification

## Abstract

Metabolomics has the potential to greatly impact biomedical research in areas such as biomarker discovery and understanding molecular mechanisms of disease. However, compound identification (ID) remains a major challenge in liquid chromatography mass spectrometry-based metabolomics. This is partly due to a lack of specificity in metabolomics databases. Though impressive in depth and breadth, the sheer magnitude of currently available databases is in part what makes them ineffective for many metabolomics studies. While still in pilot phases, our experience suggests that custom-built databases, developed using empirical data from specific sample types, can significantly improve confidence in IDs. While the concept of sample type specific databases (STSDBs) and spectral libraries is not entirely new, inclusion of unique descriptors such as *detection frequency* and quality scores, can be used to increase confidence in results. These features can be used alone to judge the quality of a database entry, or together to provide filtering capabilities. STSDBs rely on and build upon several available tools for compound ID and are therefore compatible with current compound ID strategies. Overall, STSDBs can potentially result in a new paradigm for translational metabolomics, whereby investigators confidently know the identity of compounds following a simple, single STSDB search.

## 1. Introduction

Liquid chromatography mass spectrometry (LC/MS)-based metabolomics has become an important tool in clinical and translational research. However, identification (ID) of biologically and clinically relevant compounds remains a major challenge. Depending on the sample preparation method, a metabolome may be comprised of up to thousands of unique compounds; for example, up to 4200 small compounds have been reported in human plasma [1,2]. In addition to endogenous compounds, the human metabolome contains several classes of exogenous compounds including pollutants, drugs, foods, and contributions from the microbiome [3]. Small molecules/compounds often share identical masses, making it challenging to accurately identify compounds based solely on mass and/or molecular formula [4,5]. While other technologies, such as gas chromatography mass spectrometry (GC/MS) and nuclear magnetic resonance (NMR), address many of these issues [6,7] through, for example, comprehensive GC/MS spectral libraries, many metabolomics projects are conducted using an LC/MS platform. In addition, while many tools have been developed specifically for plants, natural products, and industrial applications, these are not always applicable for human or translational studies. Therefore, this perspective article focuses on LC/MS-based clinical metabolomics and proposes a strategy to improve identification of compounds in these studies.

Identifying compounds in LC/MS studies can be difficult and metabolomics researchers have developed an array of databases, spectral libraries, novel algorithms, and other tools to address the various challenges (for review, please see [8,9]). Generally speaking, compound annotation is conducted using metabolomics databases and/or libraries. Metabolomics databases are searched using mass only or mass plus formula and include descriptors such as chemical and biological information. In contrast, MS/MS spectral libraries include data corresponding to the fragmentation of precursor molecules, whereby experimentally derived spectra are compared to spectra present in the library. Spectral libraries can contain empirical or in silico derived (i.e., computer generated) spectra. Another strategy entails the use of customized databases that may include some MS/MS spectra, including those that focus on specific sample types [10,11]. For the purpose of this perspective article, we are referring to these as sample type specific databases (STSDBs).

While the idea of STSDBs is not new, the full potential of STSDBs remains to be determined, in part because they currently exist as simple repositories of data. Our laboratory has recently developed a computational framework for STSDBs that includes scoring algorithms, thereby improving their utility, and has developed prototypic STSDBs for bronchoalveolar lavage fluid (BAL) [11] and HEK293 cells. Importantly, STSDBs rely on several currently available tools for their development and represent a complementary approach to traditional compound ID. STSDBs not only incorporate the best tools available from the field to annotate compounds, but offer a means of preserving information to improve future studies. Our experience suggests that STSDBs can greatly enhance the compound ID workflow; however, this is by no means the only solution to compound ID and concerted efforts by several groups would be required to make globally available STSDBs a reality. This article is meant to catalyze discussion regarding the challenges and benefits of this potentially field-advancing strategy.

The concept behind STSDBs is relatively simple: build a series of databases that are specific for a sample type using empirically derived data. These sample types can range from plasma or tissues to specific cell types (e.g., HEK293 cells) and from humans to rodent models. STSDBs are initially populated using a broad range of samples that represent that sample type; for example, human plasma would ideally come from males and females ranging in age, body mass index (BMI), disease state, and other clinically relevant parameters. Conversely, a cell type specific database could include data from various experimental parameters. Once databases are populated, computational strategies can be applied that enable scoring of database hits, application of false discovery rates (FDR), and quality control metrics.

For the purpose of this perspective article, a traditional compound ID workflow is compared to a proposed STSDB workflow, followed by a discussion of challenges related to traditional compound ID workflows. This is followed by a proposed framework for developing sample type specific databases (STSDBs) aimed at addressing these challenges. Finally, a discussion of limitations and advantages of STSDBs is presented.

## 2. Traditional Strategies in LC/MS-Based Metabolomics Compound ID

**Figure 1:**
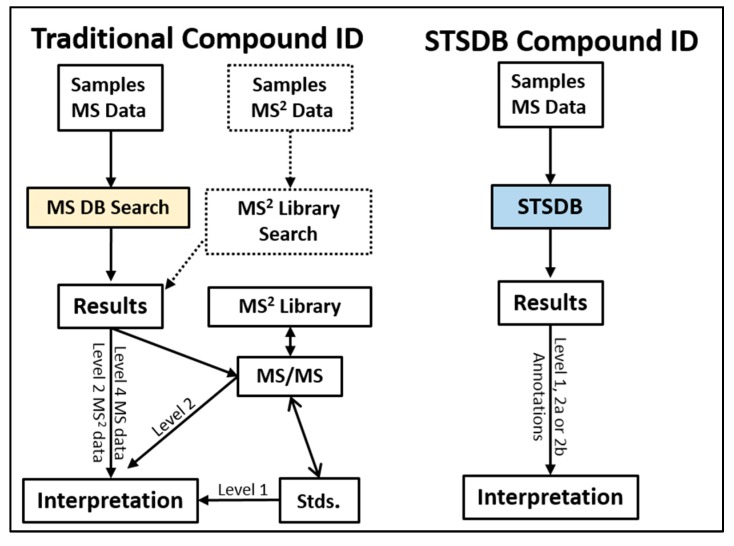
Traditional database (DB) vs. proposed sample type specific database (STSDB) searching workflows. The traditional workflow for annotating metabolomics data using databases is shown on the left. Typically, an MS database or MS/MS library search is conducted for initial annotation of compounds. Results from MS and MS/MS searches may be combined to aid in interpretation and authentic standards are used to confirm the identity of the compound. As detailed below, the confidence with which a compound is assigned a name (i.e., annotated) is currently assigned to 4 levels ranging from “unknown” (Level 4) to “Identified” (Level 1). Level 1 confidence currently requires matching to a standard (Std). Compound identification using the sample type specific database (STSDB) approach is shown on the right whereby MS data (i.e., mass, isotope ratios, and formulas) is used to search an STSDB. STSDBs store information on compounds such that higher levels of confidence are obtained following a single search.

In general, the strategy used to perform compound ID proceeds, in its simplest form, as follows: obtaining and extracting LC/MS and/or LC/MS/MS data, searching of MS databases and/or MS/MS libraries, using additional tools to interpret MS/MS spectra, and finally using authentic standards to verify compound identities [4,12] (Figure 1). With the advent of new tools, this workflow has grown more complex and more informative, however, the basic steps are essentially the same [8,9]. Unfortunately, this process is often truncated where database and/or library searches fail to provide unambiguous evidence that an annotation is correct, making interpretation difficult.

**Figure 2:**
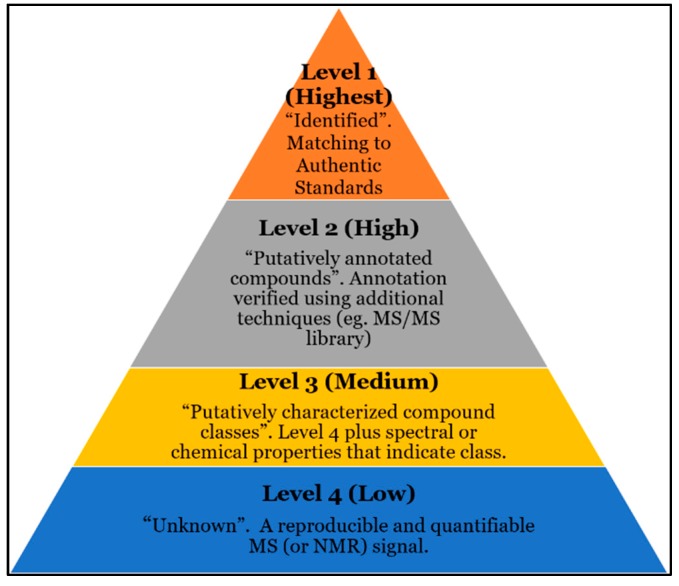
Currently accepted levels of confidence in metabolomics compound identification. The confidence with which a compound is assigned a name (i.e., annotated) is currently assigned to 4 levels ranging from unknown (Level 4, low) to putatively characterized compound classes (Level 3, medium) to putatively annotated compounds (Level 2, high) to identified metabolites (Level 1, highest). Level 1 confidence currently requires matching to a standard. Because results from previous studies are stored, including data from authentic standards and MS/MS spectra, STSDBs offer potential for Level 1–2 confidence following a single DB search.

In the context of compound annotation and identification, metabolomics research relies on “confidence levels”. “Confidence” is defined by Sumner et al. as ranging from unknown (Level 4) to putatively characterized compound classes (Level 3) to putatively annotated compounds (Level 2) to identified metabolites (Level 1) [13] (Figure 2). For simplicity, this article refers to these levels as ranging from low (Level 4) to high (Level 1). Currently, a simple MS database search will enable a user to match masses to compound names with relatively low confidence [13]. Matching an unknown spectrum to a spectrum within an MS/MS spectral library can increase confidence to a Level 2. It is important to distinguish between “annotation”, which describes Level 2–4 confidence, and “identification”, which describes Level 1 confidence. Matching an unknown’s MS/MS spectrum and retention time using authentic standards is required to reach Level 1 confidence. It is important to note that STSDBs utilize reproducible signals to increase confidence in a compound’s presence but not its ID. Rather, it should be emphasized that STSDBs currently improve the compound annotation and identification workflow.

Several tools exist for annotating and identifying compounds and no less than 30 metabolomic databases and libraries currently exist, with functions ranging from compound annotation to disease classification [14]. While MS/MS libraries are often distinct from MS databases, MS/MS spectra are also available in several MS databases; for example, the commercially available Metlin contains 16,000 MS/MS spectra with multiple collision energies per compound [15]. In addition to databases, several in silico tools have been developed that are designed to predict MS/MS fragments based on chemistry. These include LipidBlast, MetFrag, SIRIUS/CSI-fingerID, and CFM-ID [16,17,18,19,20]. For a more comprehensive review of currently available tools, please see several excellent recent review articles [8,9,21,22]. These strategies and tools are important components of a metabolomics informatics workflow and are integral to compound annotation and ID, whether using traditional workflows or in developing STSDBs.

In spite of the rich resources that are available, challenges remain. These are discussed below, following a brief description of the basic STSDB search strategy.

## 3. Basic STSDB Strategy

As mentioned, the concept behind STSDBs is to build a series of databases that are specific for a sample type using empirically derived data. STSDBs may also be referred to as spectral libraries, metabolite libraries, or personal compound database and library (PCDL, Agilent Technologies). For simplicity, we are using the word “compound” to denote molecular features extracted from the data by the feature-finding algorithm. Compounds within STSDBs are initially annotated using all currently available resources, including MS databases, MS/MS libraries, and in silico tools. A recent article by Blazenovic, et al., represents an excellent example of a prototypic STSDB, whereby the authors utilized several tools to identify as many spectra from urine as possible [10]. Completed STSDBs take this one step further by preserving the resulting dataset in a format that includes scoring algorithms to provide metrics with which to gauge the match between the unknown compound and the DB to improve utility. Within each STSDB, the search space is limited to mostly (or entirely) relevant and previously detected compounds. Importantly, separate STSDBs can be developed that represent artifacts, background compounds, and/or contaminants from LC/MS experiments. As a whole, STSDBs represent the entirety of an LC/MS experiment.

**Figure 3:**
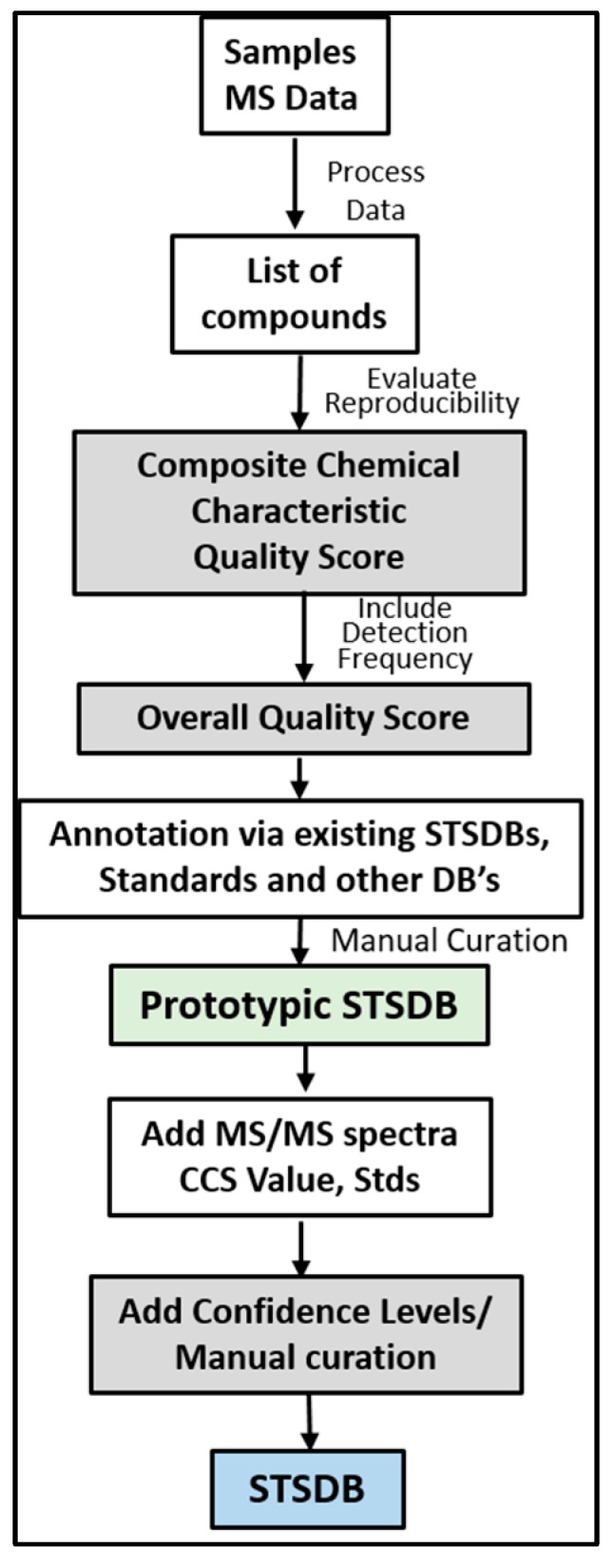
Workflow illustrating the development of STSDBs. MS data is processed and a composite chemical characteristic quality score (CCC-QS) is derived. The number of times a compound has been detected is included in an overall quality score (Ov-QS). Compounds are searched using traditional DBs to obtain initial annotations, resulting in a prototypic STSDB. Additional identifying information is added to each compound, including MS/MS spectra, collisional cross section (CCS) values, and data from authentic standards (Stds), if available. The prototypic DB is manually curated to ensure quality and confidence levels are added to each compound.

The steps of building STSDBs are fairly straightforward (Figure 3): Step (1) Conduct a comprehensive LC/MS analysis of tens to hundreds of samples representing a single biofluid, tissue, or cell type; (2) perform untargeted data extraction/mass and time alignment, including collapsing of like features, to generate a list of compounds; (3) evaluate the reproducibility of the compound and generate a composite chemical characteristic quality score (described below); (4) calculate the frequency with which the compound appears in the dataset and use this “Detection Frequency” to calculate an overall quality score (described below); (5) conduct initial compound annotation using custom databases and other databases as appropriate for the sample type; (6) perform automated curation of datasets and provide score assignments (as described below); (7) Use MS/MS and authentic standards to improve confidence in annotation and/or provide true identification. Other resources, such as NMR and ion mobility can be used if available; (8) conduct manual and automated curation; (9) continue testing, refining annotations, and flagging artifacts (repeat steps as new datasets are generated).

A major advantage of the STSDB framework is that it can include an overall quality score (Ov-QS), which is a composite score comprised of the following: composite compound characteristic quality score (CCC-QS), which takes a compound’s chemical composition and properties into account, and detection frequency (DF), which counts the relative number of times a compound has been detected across the multiple samples used to generate the STSDB. STSDBs can also include confidence ID Levels as a separate, searchable field. Within the STSDB, these scores/values can be used alone or together for filtering purposes and are stored with the database entry. If a compound rates well in all of these areas, and does not match to an artifact DB, one does not need to know what the molecule is in order to pursue it as a candidate. A compound annotated simply with a sample type and a random number, such as “compound from human liver sample_###”, for example, can still be considered an important candidate and reported as such. This is similar to a gene array, where an investigator does not need to know the function of a gene to recognize it as important in a disease. As with gene arrays, the identity and function of important candidate compounds require additional follow-up studies; STSDB scores can simply provide a filter to help determine which compounds to pursue.

STSDBs offer the ability to understand the distribution of compounds across sample types and sub-types, even before identity is confirmed using authentic standards. While current search times are short, investigators can take weeks to months moving from compound annotation to identification. Ideally, a researcher would only have to search one database/library to achieve at least Level 2 confidence. This is conceivably achieved in a single step using STSDBs through marking compounds as “previously detected” and assigning quality scores and confidence levels. An investigator simply needs to look at the output to determine if a compound match is authentic. In addition to greatly improving the speed of confident annotation and/or identification, there are several other advantages that an STSDB can offer, as described in detail at the end of this perspective article. First, we will provide an overview of some known challenges with compound ID and with some commonly used databases.

## 4. Current Challenges with Compound ID

While many excellent tools are becoming available to improve compound ID, challenges remain. For example, the time it takes to confidently ID compounds, even when using available tools, is significant. In the case of human studies, this is in large part due to the fact that these databases and tools are not entirely applicable to clinical metabolomics, where inclusion of such categories as theoretical human, xenobiotic, and non-dietary plant compounds may not be relevant. To date, it is challenging to effectively take sample preparation and instrument-generated artifacts into account, although this is changing and resources for understanding artifacts are becoming available [23,24,25,26]. Since a significant portion of a dataset may be composed of artifacts and non-biologically relevant compounds [10,27], deciphering and understanding these signals in a sample-specific manner, is vital. Finally, while studies are able to accurately annotate a larger portion of the data, preserving and fully utilizing this data remains a challenge.

The choice of database or library can also affect results, as detailed in other recent work [9,28]. For example, quality scores are currently based on chemical information only, not on the probability that a compound is actually in a sample, nor whether it can be reliably detected in an experimental system. To date, several strategies are addressing this by, for example, adding additional context to existing databases. These strategies include better cataloguing of what has been reported by the community and documentation of known compounds using online databases, including Massbank of North America (MoNA; www.massbank.us). Additional in silico-based computational tools for identification have also been developed such as MS-DIAL, LipidBlast, CSI:FingerID, and Metfrag [16,19,20,29,30,31] and applied, for example, at critical assessment of small molecule identification (CASMI) contests [28].

As described previously, to obtain a Level 1 confidence requires matching an unknown MS/MS spectrum and retention time using authentic standards [13]. While it would be challenging to obtain this information on the possible >40,000 endogenous human compounds [32], this is becoming more and more possible with the development of comprehensive MS/MS spectral libraries and searching tools and the reader is encouraged to see [33] for review. However, these resources do not yet take sample-type specific information into account and mis-annotation remains an issue. In addition, the exclusive use of authentic standards to confidently ID compounds also assumes that the standards are available for the compounds of interest. As mentioned above, a significant portion of a dataset may be due to “noise”, including fragments [27], background contaminants, and sample prep artifacts, for which standards are neither available nor helpful. While STSDBs do not offer simple solutions to all of these challenges, they represent a step in the right direction.

## 5. Challenges with Current Databases

One major challenge with searching non-specific databases is that searching using only neutral mass values usually results in ambiguous matches where over 100 compounds may match to a single neutral mass [4]. This issue is not fully resolved with the addition of isotope ratios and formula generation, nor even by high resolution MS [4,5,34]. A second challenge lies in the fact that the human metabolome encompasses both endogenous compounds and compounds from the exposome [35], adding to the challenge of filtering through multiple database matches. The exposome essentially comprises all exogenous compounds to which humans can be exposed, including pollutants, foods, beverages, drugs, and airborne plant and animal particles [3,15,36,37,38]. While it is unclear how many exposome compounds can actually be detected in humans using LC/MS-based metabolomics profiling, their relevance to human health is potentially significant [3]; therefore, informatics strategies must take these into account. Unfortunately, current strategies entail searching very large databases in an attempt to match all possible endogenous and exposomic compounds; this results in artificially large search spaces and an increase in false annotations [4]. In fact, many/most of these generic database entries are not even found in the sample type being studied, leading to extensive manual curation of the data. Fortunately, tools are available to assist with this, such as fields within HMDB that indicate if a molecule has been previously detected and/or quantitated in humans. Smaller, customized databases have been proposed as a solution to decrease false annotations [4,5,39]. This reinforces the idea that STSDBs can not only reflect differences in metabolomes across sample types, but could potentially be used for semi-quantitative comparisons.

Current metabolomics DBs are extremely informative, with up to 100 descriptors available for each compound [32]. These include compound names, chemical class, biospecimen type, diseases, and links to other resources. Most databases allow for constrained searching based on some of the above descriptors; while this results in smaller search spaces, it does not address the issues of annotation errors and low confidence annotations. While some information is available on which biospecimen(s) the compounds have been detected, manual curation is still generally required to obtain this information. In addition to knowing where a compound has been detected, information regarding the frequency of detection in a given sample type could greatly aid in confidently determining if a given compound is found in that sample type. When incorporated into a quality scoring algorithm, as is possible with STSDBs, this information can be used to determine the likelihood that a compound is present in a given sample type.

Lipids are a unique challenge in MS-based databases because it is impossible to determine exact species based on neutral mass alone. For example, a mass of 835.6091 may match to PC (20:2(11Z,14Z)/ 20:3(5Z,8Z,11Z)) in a simple database search. However, it would be more appropriate to label this as PC (20:2/20:3), or even as PC (40:5), unless high resolution MS^n^ data can distinguish the location of double bonds. Level 1 confidence will be difficult to obtain for some lipids even with authentic standards [40,41]. More precise identification of specific lipids using a variety of solutions unfortunately is unlikely to be achieved within the current DB frameworks. Conversely, STSDBs may offer improved lipid annotations due to the fact that once a mass is confidently annotated or identified within a dataset, its annotation will not change.

## 6. Challenges with Current Focused DB Approaches

While widely used metabolomics databases are rich in information and essential to the metabolomics informatics workflow, clearly there are challenges that render them less than ideal for rapid and confident compound annotation and ID. Conversely, the concept of focused DBs is beginning to take hold [4,39,42]. Importantly, STSDBs are based on existing resources and yet fill a major void, namely the systematic organization of databases so that searches can be conducted in a focused and study-specific manner. This has been recognized in genomics for almost a decade, where tools such as the Tissue-specific Gene Expression and Regulation (TiGER) and Tissue-Specific Genes Database (TiSGeD) comprise large scale repositories for analyzing data on tissue-specific gene expression and regulation in a variety of human tissues [43,44]. Similarly, the Human Proteome project categorizes peptides and proteins in a sample type specific-related manner [45].

Examples of strategies in metabolomics that are similar to STSDBs include the HMDB-supported serum [1], urine [46], salivary [47], and cerebral spinal fluid (CSF) [48] databases. The newly released “BinVestigate” at the West Coast Metabolomics Center (WCMC) is a GC/MS-based tool that includes information regarding sample and disease types, with tools to visualize where compounds have been detected. “creDBle [27]” comprises an *E. coli*-specific resource and the equivalent of a urine STSDB was presented by Blazenovic et al. [10]. These are some of the first studies, to our knowledge, that attempt to describe 100% of the data within a dataset. Support for the development of STSDBs is offered through the Model Organism Metabolomes (MOM) task group of the Metabolomics Society [39] and through efforts towards a tissue-specific *Drosophila melanogaster* Atlas [42]. While a definitive step in the right direction, several issues must be overcome before such tools are effective and adaptable by the entire clinical metabolomics community. Because the HMDB sub-databases focus on human samples and include some LC/MS data, we will focus our attention on these.

In our experience, the HMDB biofluid DBs are very useful to understand what compounds have been detected in a particular biofluid using a variety of technologies and strategies. For example, they include data from ICP-MS, NMR, and targeted MS/MS runs. However, compounds detected by these methods, such as lipid mediators and metals, may not be measurable by typical LC/MS-based metabolomics methods, which is the focus of this perspective article. In addition, they do not include scoring metrics that can be used to determine the quality of the DB entry and the likelihood that a compound is present in a given sample type. They are also limited in the number of samples analyzed to populate the databases and do not stratify the relative quantities observable in various cells or tissues. Finally, these databases do not attempt to understand 100% of the datasets under study.

For example, while the HMDB CSF database includes 476 compounds that have been confirmed to exist in human CSF, the DB was generated using seven samples from adult Caucasians [48,49]. Importantly, only 17 of these compounds (3%) were detected using LC/MS, the remaining 97% of compound identifications were generated using GC, NMR, and targeted methods. Other search strategies are required to annotate or identify the remaining MS peaks in a CSF LC/MS experiment. In addition, HMDB reports 4229 “confirmed and highly probable” compounds found in human serum, with only 96 of these from targeted LC/MS/MS analyses, and none from LC/MS profiling methods [1]. The HMDB urine and saliva databases have no compounds detected with an LC/MS method [46,47]. While clearly important and valuable information for metabolomics researchers, we submit that expanding these databases into an STSDB format could offer a more comprehensive resource specifically for clinical LC/MS studies.

The currently available HMDB-biofluid, BinVestigate, and creDBle [27] databases likewise represent a step in the right direction. To further expand and improve on these concepts so that STSDBs can be widely used by the clinical metabolomics community, several things must happen. First, we need to develop databases that represent a wider array of clinically relevant sample types. Second, these databases must be populated by large numbers of samples from a range of disease and non-disease states. Third, datasets must be automatically and manually curated to annotate as close to 100% of peaks as possible. Fourth, informatics tools must be developed and included that will allow investigators to make full use of these unique and valuable resources. Our experience suggests that all of these are possible, although a concerted effort will be required. The following section describes a framework for achieving the first step.

## 7. Framework for Developing STSDBs

**Figure 4:**
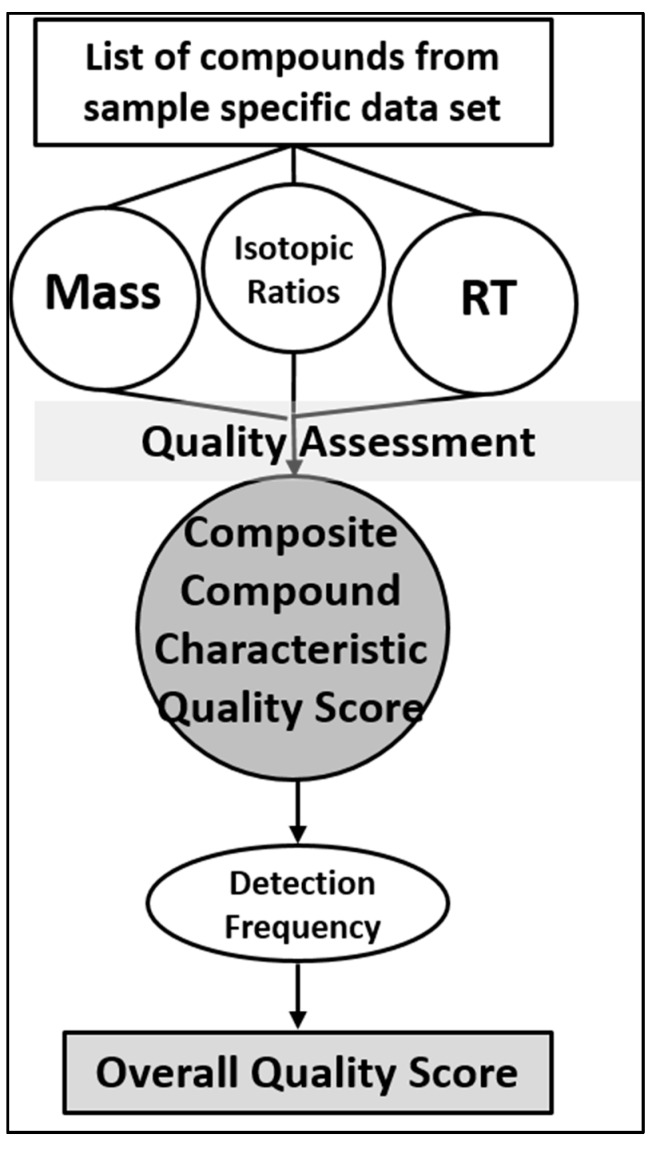
Derivation of the quality scores (QS). MS data are initially assessed for quality within a dataset using standard metrics such as variability in mass, isotope ratios, and retention time. This is used to generate a composite compound characteristic quality score (CCC-QS). The number of times a compound is detected in a sample, i.e., the detection frequency, is included in a final overall quality score (Ov-QS).

STSDBs include all of the strengths of general databases, including compound-specific information and quality scores. While the concept of STSDBs is not entirely new, the newly proposed design and features of STSDBs may make them extremely attractive to the community. As mentioned, STSDBs should provide an improved compound annotation and ID workflow through an overall quality score (Ov-QS- Figure 3 and Figure 4), which includes a composite compound characteristic quality score (CCC-QS) and relative detection frequency (DF), which counts the number of times a compound has been detected in a given STSDB. A description of these and the overall utility of STSDBs is demonstrated through our prototypic STSDBs, which were recently developed for BAL [11] and HEK293 cells. We are using the term “prototypic” because these STSDBs have not yet been fully curated and the datasets used to populate these DBs are limited.

## 8. Prototypic STSDBs for Bronchoalveolar Lavage (BAL) and HEK293 Cells

Our team has already assembled and tested a prototypic BAL-DB using mouse and human BAL fluid samples [11]. Data processing followed a typical workflow and included additional steps for database assembly (Figure 1 and Figure 3). The prototypic BAL database contains a comprehensive list of reproducibly detected compounds from lipid extracts in positive and negative ionization mode (3163 and 1330 entries), and the aqueous extract in positive ionization mode (689 entries). This prototypic DB did not include merging or consolidation of positive and negative data, rather they were treated as separate experiments. Analysis of BAL fluid from an independent study acquired one year later produced a match rate of 81% to BAL-DB when search constraints of 10 ppm mass accuracy and 1.5 min RT were used. The large retention time window was used to account for the variance in chromatography between the two experiments which were conducted 1 year apart. This can be compared to 20% annotation when Metlin was searched with 10 ppm mass accuracy and no RT. Importantly, the STSDB format allows for information from studies to be preserved. So as more and more MS/MS spectra and data from authentic standards are included, more BAL-DB entries will match with a ≥Level 2 confidence. This assumes that all compounds will produce high QSs; importantly, it also demonstrates the potential when information is stored as a STSDB.

**Figure 5:**
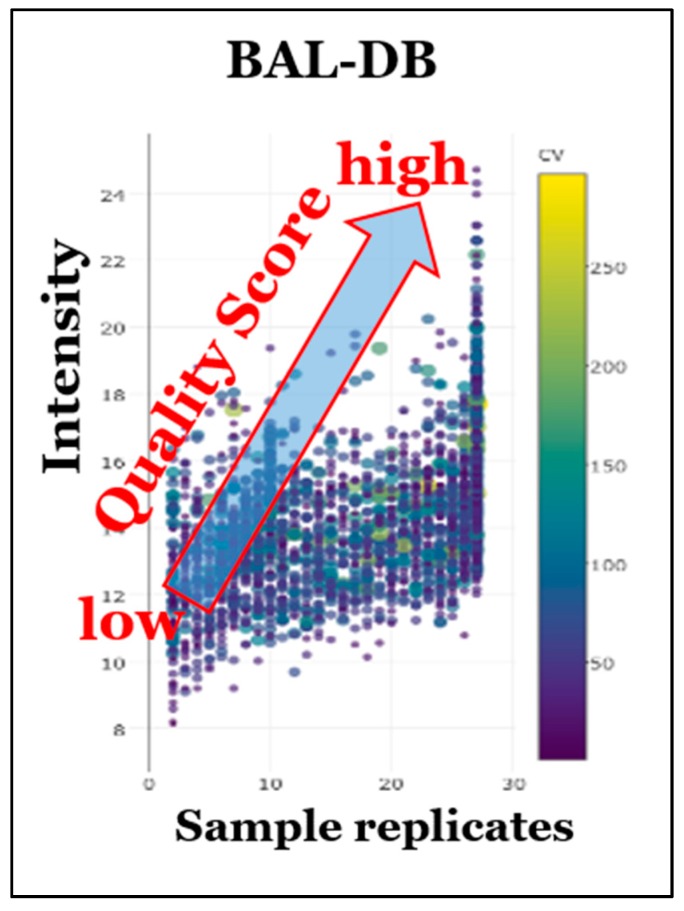
Distribution plot of prototypic BAL-DB entries. This figure illustrates how CCC-QS increases as DF and intensity increase. Samples with a low number of detections and low intensity will have overall low QS, as seen in the bottom left of the plot. Conversely, a compound that is detected in all samples with high abundance will have a high QS, as seen in the top right of the plot. The colored bar on the right indicates the inverse coefficient of variability (CV), with yellow being a high %CV and blue being a low %CV. Even low intensity compounds will score higher if their DF is high.

Our BAL-DB demonstrates the utility of our proposed relative detection frequency (DF) statistic. As mentioned, the DF counts the number of times a compound has been detected in a given STSDB and relates this to the total number of samples surveyed in that STSDB (i.e., number of times detected/number of total samples). As expected, the more samples that are analyzed, the more compounds demonstrate a high DF. For example, Figure 5 illustrates how the QS will increase relative to the trends seen in the STSDB (Figure 5). Compounds with lower DFs will have lower quality scores (QS); the more times a molecule is detected, the higher the DF and the higher the QS. This also verifies that the data processing steps aligning the compounds by RT and mass are effective. In conclusion, we have already developed a searchable human and mouse BAL STSDB; however, the current version is prototypic and therefore does not include the features of a full STSDB.

One main advantage of STSDBs over conventional DBs is the ability to develop computational methods to score the quality of database entries. This information is stored in the STSDBs and updated as new data is added. Evaluation of compound characteristics, including mass (M), isotope ratios (I), and retention time (RT), is typically achieved through calculating intra-and inter-experiment coefficients of variation (%CVs). When current databases are used, these CV’s are considered separately for each compound characteristic. With STSDBs, a single, “composite chemical characteristic” quality score (CCCS-QS) can be useful to help determine if an entry in the database is potentially relevant to a study. Together, the CCS-QS and DF comprise a composite Overall Quality Score (Ov-QS). Overall, three criteria must be met for a compound to be considered authentic: (1) It must have a high Ov-QS score, (2) MS/MS, CCS, or other data must support its annotation, and (3) the compound has not been found in an artifact/contaminant DB. Importantly, once this information has been generated and preserved for a compound, it is immediately available following a DB search.

## 9. STSDB Computational Strategies

Currently no algorithm addresses the reproducibility of compounds in a database. Rather, common statistical methods are utilized to explain each compound’s characteristics (mass, isotope ratios) separately. We propose a scoring metric that combines these properties into a single score, the CCC-QS. We then include DF to produce an Ov-QS. This will represent a major improvement to the workflow by introducing algorithms that confirm each individual compound’s annotation following a single database search and measures the frequency of its presence in each STSDB. Rather than rigorous validation post hoc to data collection, detected compounds are stored as previously detected and/or validated compounds with reliability scores.

**Figure 6:**
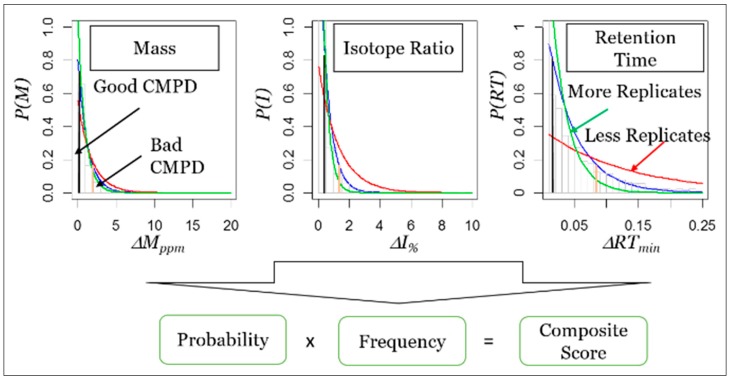
Distributions of M, I, and RT from our HEK DB are shown by blue lines. Red shows theoretical trends with fewer replicates, green shows trends with more replicates. Black and beige verticle lines show how a P value represents a “good” and “bad” compound.

These scores are updated as more evidence is collected for each compound. The CCCS-QS will be computed using the measured characteristics (RT, mass, isotope ratios) using computational methods that are generalizable across a number of challenges, including those developed by our co-author [50,51]. Composite compound characteristic quality scoring algorithm: The compound characteristics of mass (M), isotope ratio (I) of the monoisotopic and M + 1 peak, and RT are used in a novel algorithm to score the reproducibility of a compound. For our purpose, Δ*M*, Δ*I* and Δ*RT* are the measured differences between the compound’s mean and replicate values. The Δ*M*, Δ*I* and Δ*RT* of each replicate of a measured compound is modelled to statistically measure the chance that each replicate is from the same compound (Figure 6). From these modeled distributions, the compound composite probability is computed from the independent probabilities for each compound (Equation (1)).

(1) $$P_{i}=P\left(\Delta M_{i}\right)*P\left(\Delta I_{i}\right)*P\left(\Delta RT_{i}\right)$$

(2) $$S_{i}=n_{i}/N*P_{i}$$

Each probability is then weighted by the relative detection frequency (DF) of replicate observations and then the final composite score *S* is computed for each compound (Equation (2)).

A composite score ranges from 0 to 1.0, where 1.0 would represent a perfectly reproducible compound that is detected in every sample. Finally, Bayesian updating of the models and scores are used as new replicate data is acquired. After updating, the compound composite scores $S_{i}$ are computed as the product of the *P_i_* determined from these posterior distributions and the manually updated *n_i_* and *N* values. Because of the exponential distribution, the final score for each compound is always on a scale of 0–1.0. The method uses compound reproducibility, which is weighted according to how many replicates have been observed, together with the other described scoring components, and updates the database as new samples are acquired and added. Figure 6 shows distributions of M, I, and RT for our prototypic HEK-DB (blue lines) where more replicates result in higher quality scores.

## 10. Limitations of STSDBs

While STSDBs have tremendous potential, there are also limitations. Although inclusion of RT is used in the development of STSDBs, through the calculation of the CCC-QS, importantly RT is not required to search the STSDBs. Undoubtedly inclusion of RT will improve annotation results. However, the added value of RT in the search of STSDBs is still unknown. Importantly, STSDBs reduce the search space so the likelihood of obtaining multiple matches for the same compound is lower than it would be if conventionally large databases are used. Unfortunately, we are not able to fully evaluate this variable until algorithm development has been completed and statistics can be generated. However, compounds with multiple unresolved DB matches can be flagged and given a higher priority in obtaining orthogonal identifying information. Finally, datasets and STSDBs can be used to develop and test RT indices, whereby the order in which a molecule elutes can be taken into account [52]. RT indices will allow users the benefits of RT even when LC methods different than those used to create the STSDB are used.

It should be noted that routinely used sample preparation and LC/MS methods will not allow for the detection of all compounds. We address this in our own lab by developing targeted methods for compounds (e.g., oxylipins, nucleotides [53,54]) that are not typically detected in our LC/MS profiling studies. The purpose of STSDBs is not to provide 100% coverage of a particular metabolome; rather, if a compound can be detected using standard methods, it will become part of an STSDB. In other words, the purpose is to create databases of compounds consistently detected using standard LC/MS methods.

It is also important to recognize that Level 1 ID still requires matching to authentic standards. It is unknown how close an investigator will come to confidently annotating 100% of the extracted dataset when using an STSDB. It is important to again note the difference between “annotation”, which describes Level 2–3 confidence, and “identification”, which describes Level 1 confidence. While not exclusively an issue with STSDBs, the identification of isomers is an additional challenge. Based on an evaluation of prototypic *STSDBs* for BAL, plasma, HEK293, and HaCaT cells, we find that even with a prototypic STSDB, which has not yet undergone manual curation, we would obtain high (>0.90) Ov-QSs for 75% of compounds. Subsequent versions will have fewer Level 3 and more Level 2 annotations as manual curation and MS/MS data are incorporated. Because they are reproducibly detected and manually validated database entries, we argue that compounds with high Ov-QSs are “real” compounds, even if they have not been assigned a compound name. In the long run, this may result in modified confidence levels, an effort that is already underway [9].

## 11. Advantages of STSDBs

There are several ways STSDBs provide advantages to traditional compound ID strategies. For example, the improved rate of identification when focused databases are used has already been demonstrated using the HMDB serum and urine databases [1,46] and creDBle [27] again, this is because once a compound’s identity has been verified using authentic standards, it is always identified in the STSDB. The resulting reduction in search space dramatically reduces the rate of false positives. Conversely, searching can be conducted in an iterative fashion across several STSDBs and any compounds that do not match an STSDB can be searched using other DBs and libraries; therefore, the issue with “false negatives”, is minimal. Our BAL STSDB [11] also supports an improved identification rate using STSDBs.

STSDBs also allow investigators to confidently annotate the most common unknowns, so that clinically relevant, but unknown, compounds are still pursued. For example, if a molecule that has been detected in >80% of a dataset comprised of 200 liver samples produces an interpretable MS/MS spectrum when fragmented, and is not an artifact, a researcher can be reasonably sure the molecule is part of the liver metabolome, even if he/she cannot definitively identify it. In addition, once that compound is identified, results can be uploaded into the STSDB, improving future results. Finally, STSDBs enable researchers to reduce the number of possible identities for the uncommon unknowns, largely by reducing the effective search space.

In addition to focusing on biofluids and cells, STSDBs can be developed for any number of purposes. For example, searching a “contaminant” DB can quickly determine if compounds are biologically relevant or due to processing. Similarly, an “in-source fragment” DB can be developed for a specific instrument platform and shared amongst users. These cannot be based on theoretical or in silico data, they require empirically-derived datasets. Indeed, as mentioned previously, resources such as these are becoming available [24,39]. These types of databases are also critical to the field of metabolomics as a whole, where such DBs can be cross-checked by any metabolomics laboratory. Finally, STSDBs represent a convenient means of sharing information amongst users with either instrument platforms, biospecimens, or disease interests in common. For example, an STSDB could be developed for cancer, with corresponding fields to indicate the type of cancer where a compound has been detected. Overall, the development of STSDBs can be considered an important first step towards the development of a Human Metabolome Atlas, whereby compounds can be compared across tissues/cells.

STSDBs can make manual annotation of datasets less daunting. While manual curation seems impossible for a set of 50,000 theoretical compounds, manual curation is achievable for STSDBs, especially if conducted by multiple groups. Our prototypic BAL database contains <5000 compounds, which can be manually curated to produce a final STSDB. More fully populated and curated STSDBs will contain whatever number of compounds are reproducibly detected in that specific sample type. After that, it is relatively straightforward to add compounds to the database as more samples are added and more compound identities are established. Community members can aid in this effort, as demonstrated by the widely used the Global Natural Products Social Molecular Networking tool [55].

Building a series of STSDBs can also greatly expand our understanding of non-biological LC/MS peaks. This includes acquisition artifacts such as in-source fragments, and data extraction artifacts such as incorrect mass assignments caused by incorrectly assigned charge carriers. These also include ions introduced from reagents and materials used in the sample preparation process. Knowing the identity of these compounds will enable researchers to rapidly eliminate them from their list of compounds. Again, this can be accomplished using a series of “artifact/contaminant/fragmentation databases”.

Finally, STSDBs allow for FDR strategies to be applied. For example, in proteomics, FDR for peptide identification relies on the fact that protein databases are well curated and relatively static. FDR is achieved by reversing the sequences in these databases and searching data against this presumably false, reverse-databases. Once an STSDB has been developed, FDR tools can be built, for example, based on compounds that are not in the database or not biologically plausible. The development of such tools has begun to be explored in metabolomics [56] and others have already proposed that small search spaces can improve annotation [5].

## 12. The Way Forward

While STSDBs appear to offer significant advantages over conventional DB searching strategies, the actual development of these resources faces its own set of challenges. For example, the extensive funding required for building a series of STSDBs would likely have to come from multiple sources; as such, a global effort by multiple laboratories is likely required. One may legitimately ask why an investigator cannot simply submit more experimental data to existing DBs. As mentioned, expansion of the HMDB-biofluid DBs to include scoring algorithms is one way forward that seems feasible and one we personally hope gains support. Alternatively, it has been suggested that additional metadata could be uploaded into other, general DBs. This is potentially feasible, although it may be challenging to integrate the proposed scoring algorithms into general databases. Further, the main advantages of STSDBs stem from the fact that they comprise constrained spaces, where manual curation, near-complete interpretation of an LC/MS run, FDR, and RT indices are made more feasible. While it may be possible to restructure existing DBs, they too have considerable value as-is and it seems that the community could benefit from both strategies.

Regardless of the actual implementation strategy, resources would be required to conduct sample preparation, analysis on multiple LC/MS platforms, and initial data analysis; this would be needed for each STSDB developed. Similarly, it would be necessary to develop and maintain infrastructure and standards for building, storing and accessing STSDBs. Finally, acquiring high quality and well-characterized samples would require the collaborative efforts of several clinical groups. This in itself would require efforts aimed at standardizing methods. Funding agencies and international/national organizations could conceivably provide the forward momentum necessary to get started. As an international affiliation with dedicated task groups, the Metabolomics Society is perhaps ideally situated to provide support, e.g., through a forum, for such an effort. While limitations clearly exist, we submit that STSDBs still provide not just a step, but a dramatic leap in the right direction.
